# Retrieving a displaced third molar from the infratemporal fossa: case report of a minimally invasive procedure

**DOI:** 10.1186/s12903-019-0852-z

**Published:** 2019-07-15

**Authors:** Jean-Christophe Lutz, Roberto Luigi Cazzato, Marc-Kevin Le Roux, Fabien Bornert

**Affiliations:** 1 Oral and Maxillo-Facial Surgery Department, Strasbourg University Hospital, 1, avenue Molière, 67098 Strasbourg cedex, France; 2 Faculty of Medicine, University of Strasbourg, 8 rue Kirschleger, 67000 Strasbourg, France; 3 INSERM (French National Institute of Health and Medical Research), “Regenerative Nanomedicine” laboratory, UMR 1260, Faculté de Médecine, 67085 Strasbourg Cedex, France; 4 Department of Interventional Radiology, Strasbourg University Hospital, 1, place de l’Hôpital, 67091 Strasbourg cedex, France; 5 Oral and Maxillo-Facial Surgery Department, Marseille University Hospital, 147 Boulevard Baille, 13005 Marseille, France; 6 Department of Oral Medicine and Oral Surgery, Dental Clinic, Strasbourg University Hospital, 1, place de l’Hôpital, 67091 Strasbourg cedex, France; 7 Faculty of Dentistry, University of Strasbourg, 8 rue Sainte Elisabeth, 67000 Strasbourg, France

**Keywords:** Surgery, Image-guided, Molar, third, Complications, Radiology, interventional

## Abstract

**Background:**

The appropriate management of postoperative complication of wisdom teeth removal is of utmost importance as it can result in legal procedures.

The accidental displacement of a maxillary third molar in the infratemporal fossa (ITF), is a rare complication that can occur even with experienced surgeons.

The numerous retrieval techniques reported are invasive and provide an unpredictable access.

Our aim was to achieve the safe and swift retrieval of the tooth displaced to an area of such complex anatomy.

**Case presentation:**

We describe the case of a 17-year-old female patient whose right upper third molar was accidentally pushed upward to the ITF and became unreachable.

Retrieval based on interventional radiology using the CT-guided placement of a bone trocar above the displaced tooth was successfully performed. The postoperative course was uneventful.

**Conclusions:**

CT scan assisted interventional radiology provides both, real-time assessment of the tooth position through image refreshment, and steady stabilization of the displaced tooth. Therefore, it allows a safe and non-traumatic retrieval with a time-efficient procedure achieved through a minimally-invasive approach with inconspicuous scaring. We believe that such a procedure is an interesting treatment option for optimal outpatient care.

To our knowledge, no such case has been previously described.

**Electronic supplementary material:**

The online version of this article (10.1186/s12903-019-0852-z) contains supplementary material, which is available to authorized users.

## Background

Postoperative complication of wisdom tooth removal greatly deteriorates patient’s experience and can result in legal procedures, especially as there is insufficient evidence on whether or not such asymptomatic disease-free teeth should be removed [1]. The appropriate management of such complications is therefore of utmost importance.

Maxillary third molars can be accidentally displaced into a variety of locations including the buccal space, infratemporal fossa (ITF), maxillary sinus, or other tissue planes [2, 3].

The incidences of displacement into the ITF are unknown because this complication is not mentioned in large prospective series [4], but only through case reports [5–8]. This complication can be prevented with a distal retractor [2], but may occur occasionally even with the most experienced surgeons.

Extraoral and intraoral retrieval techniques have been reported [6, 9–13], sometimes using additional instruments. Exploratory blunt dissection in the ITF can be dangerous because of its complex and rich anatomy and can result in additional complications.

Swift retrieval, combining accurate localization and safe approach, is advisable to avoid deteriorating patient experience that is already impaired by a surgical complication.

Our aim was to describe a technique based on minimally invasive interventional radiology. It provided a safe and swift retrieval of a displaced third molar by image guidance and trans-oral trocar-assisted tooth stabilization. To our knowledge, no such case has been previously described in literature.

## Case presentation

**Fig. 1:**
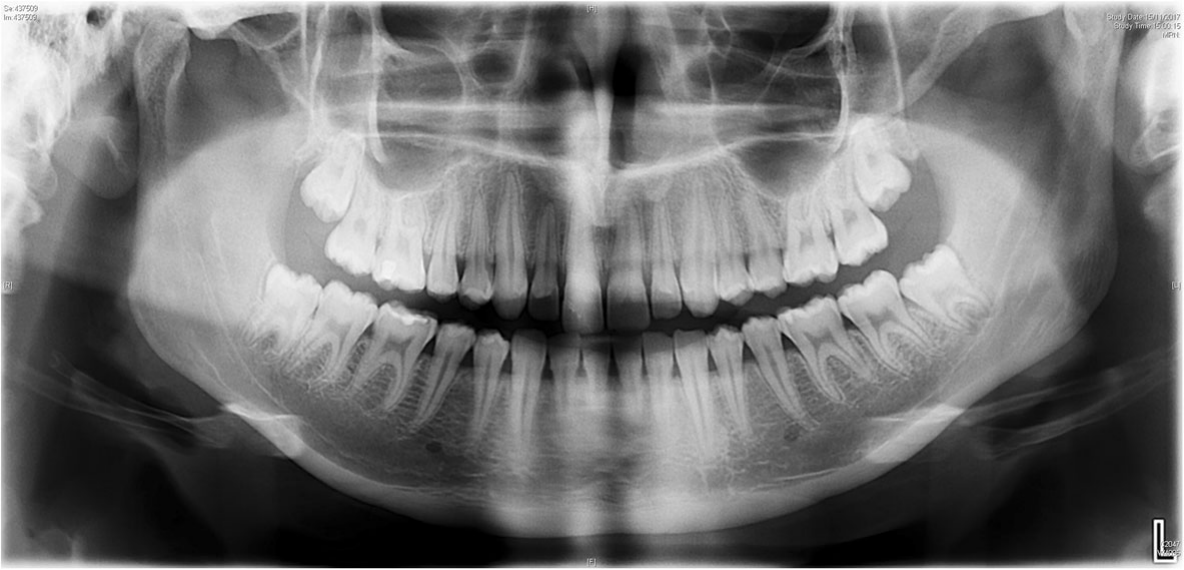
Preoperative panoramic radiograph

A 17-year-old female student was referred to the Department of Dentistry for the prophylactic removal of 4 unerupted third molars due to dental crowding. She had no medical history. Teeth extraction was planned in two sessions under local anesthesia (Fig. 1).

The extraction of the left upper and lower third molar teeth was uneventful. During the second step 2 weeks later, the right upper third molar was accidentally pushed upward and laterally and became unreachable. Immediate retrieval maneuvers were unsuccessful. The wound was closed using 3/0 polyglactin sutures, and amoxicillin–clavulanate (1000 mg × 3/day) was administered orally for 3 weeks.

**Fig. 2:**
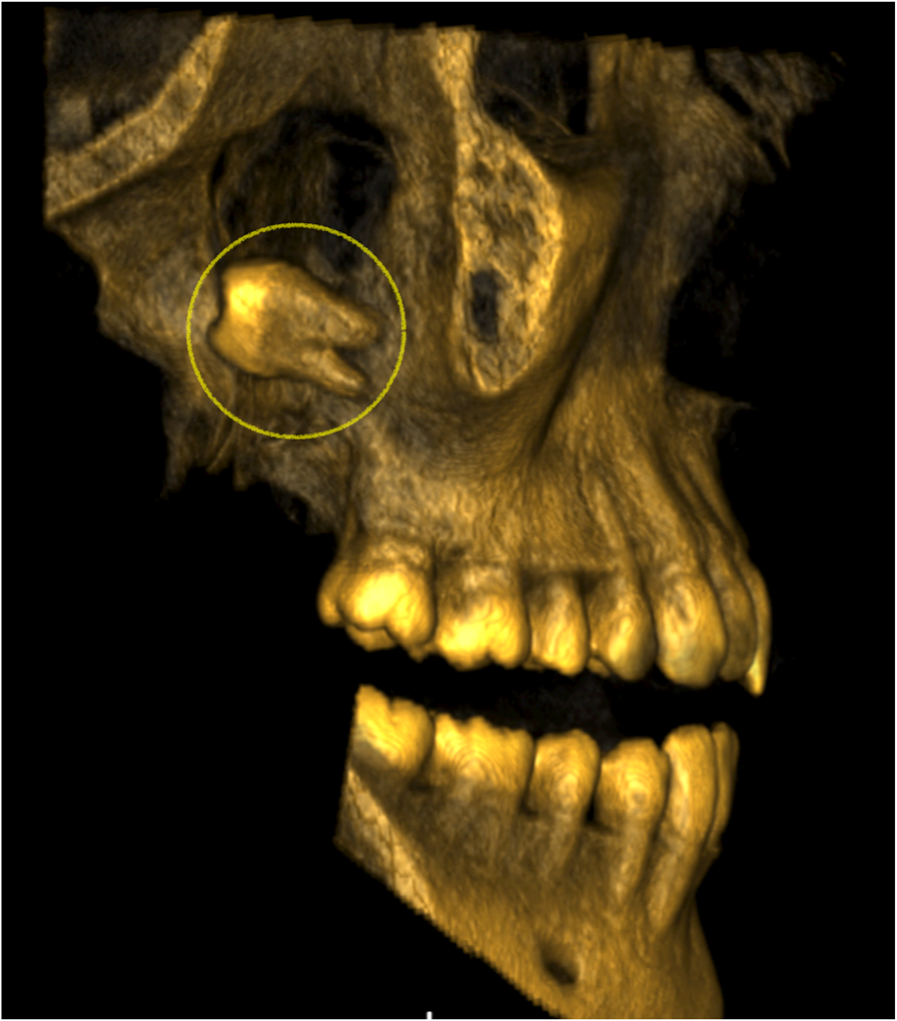
Immediate postoperative CBCT: 3D reconstruction removing the mandible and the zygomatic arch showing the displaced right upper third molar in the infratemporal fossa (circle)

An immediate postoperative cone beam computed tomography (CBCT) scan showed displacement of the third molar to the ITF up to the level of the sigmoid notch of the mandible (Fig. 2). The position of the tooth was horizontal, with the crown being posterior (Fig. 2).

**Fig. 3:**
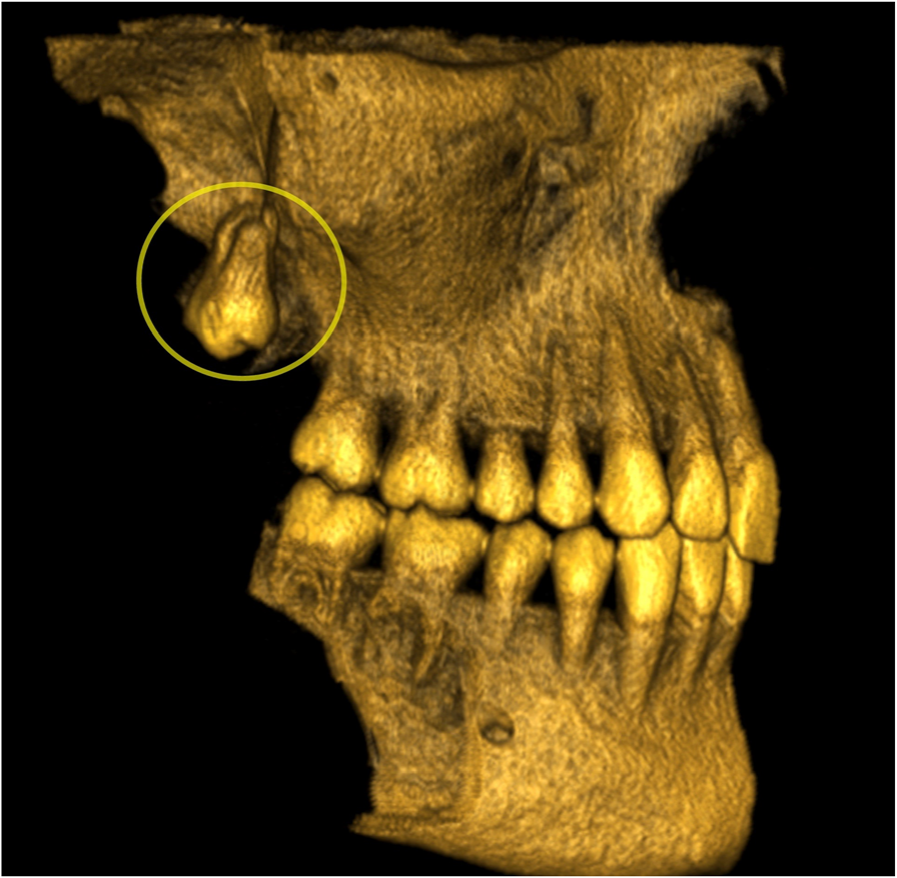
Late postoperative CT scan after 3 weeks: 3D reconstruction removing the mandible and the zygomatic arch showing downward migration of the displaced right upper third molar (circle) down to the level of the lingula

A computed tomography (CT) scan acquired 3 weeks after the procedure showed that the tooth had spontaneously returned to the vertical position with a downward crown and had migrated back down to the level of the lingula (Fig. 3).

The lack of a solid cranial support of the tooth observed on the CT scan led to the patient being at risk of further upward displacement during a retrieval attempt. A combined surgical and image-guided approach was decided.

A retrieval procedure under general anesthesia using nasotracheal intubation was performed in the interventional 4D MSCT scan unit, 2 months after the initial extraction attempt because of organizational purposes (Additional file 1). Indeed, the patient was unavailable and, as she did not complain from any significant symptoms, there was no emergency for retrieval.

**Fig. 4:**
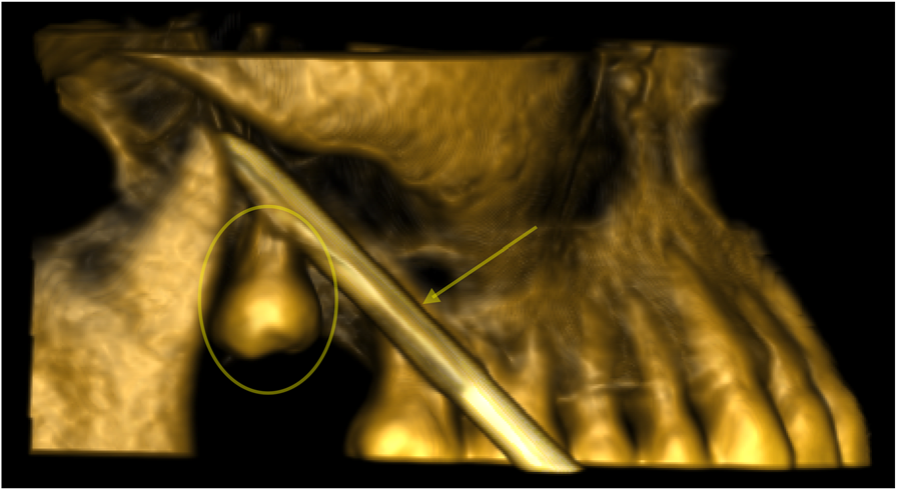
Intraoperative CT scan: 3D reconstruction confirming the position of the trocar (arrow) above the right upper third molar (circle) and between its apices, providing cranial stabilization

In the first step, radiologists inserted a 12 Gauge bone trocar (Bonopty®-AprioMed AB, Sweden) under CT-guidance (Toshiba MEC Aquilion ONE®) through the superior buccal sulcus by an ascending approach. The trocar was positioned exactly between the tooth apices to provide cranial support (Fig. 4).

The extraoral end of the trocar was then gently tilted upward to push the crown downward. Finally, after marking the parotid duct papilla and infiltrating the mucosa using adrenaline and epinephrine 1%, an incision was performed through the posterior superior buccal sulcus.

**Fig. 5:**
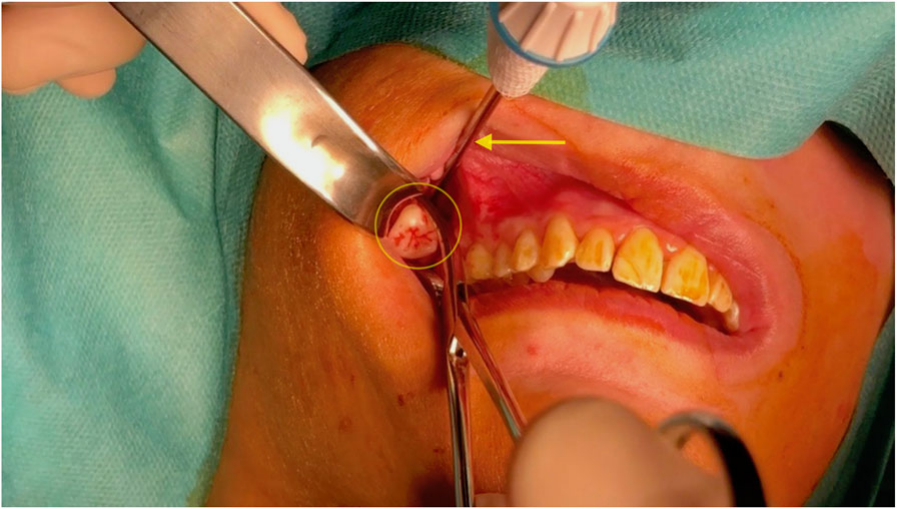
Removal of the displaced wisdom tooth (circle): The trocar (arrow) has been inserted through the right posterior part of the superior buccal sulcus, where a small incision was performed to allow intraoral tooth retrieval using a pair of Kelly forceps

The crown was identified and easy retrieval using a Kelly forceps was achieved (Fig. 5). The right lower molar was extracted simultaneously. The surgical approach was closed using 3/0 polyglactin absorbable sutures, and postoperation, amoxicillin–clavulanate (1000 mg × 3/day) was administered orally for 3 weeks.

The CT-guided insertion of the trocar in the appropriate position took a total of 19 min. The surgical retrieval took 3 min. The total dose-length Product was 284.00 mGy.cm. The patient did not feature any significant edema, pain, or mouth opening limitation after 6 h and was discharged, therefore achieving outpatient care. The patient had no edema and was very satisfied with her surgical management. Postoperative follow-up at 3 weeks and 1 year did not retrieve any complication.

## Discussion and conclusions

The displacement of a maxillary third molar to the ITF is a well-known complication of wisdom tooth extraction [6, 14].

The ITF features an anatomical complexity because of the maxillary artery, the venous pterygoid plexus, the sphenopalatine nerve, and the pterygoid muscles [15]. It is also a deep region of the face, with difficult access to it. Therefore, the retrieval of a displaced wisdom tooth is both hazardous (due to hemorrhage and nerve damage) and uneasy (due to further upward migration toward the skull base).

Tooth retrieval is recommended because of potential secondary complications such as infection, limited mandibular motion, or psychological unease.

The appropriate timing for retrieval ranges from immediately to 4 years after the displacement, wherein a majority of authors proceed from the first month [6].

In the present report, the displaced right upper third molar had spontaneously migrated downward and straightened, according to the control CT scan acquired after 3 weeks (Fig. 3). This suggests, in accordance with literature [15], that there is no hurry for retrieval, as natural chewing movements and fibrosis [16] seem to bring the tooth back to its original position.

**Conventional retrieval procedures use an intraoral approach** through a long incision in the superior buccal sulcus, thereby exposing the posterior maxilla. They are inconsistently successful [9], as they do not provide a predictable access to the displaced tooth [6].

Proposed alternative techniques used either a single intraoral approach or a combined intraoral-extraoral approach, sometimes utilizing additional devices [6].

Most of the **alternative intraoral techniques** use a trans-sinusal approach requiring two osseous windows made through, respectively, the anterior and posterior walls of the maxillary sinus [17]. Despite the trans-sinusal approach being considered as a method of choice [10], it does not facilitate the placement of an instrument to stabilize the tooth cranially or posteriorly. It also causes tissue trauma, thereby exposing the patient to facial edema and secondary emphysema.

**Extraoral approaches** either directly retrieve the tooth or stabilize it cranially to facilitate intraoral retrieval. Several techniques approach the ITF from the top by using either a coronal [11] or a Gillies temporal incision [12] or inserting a spinal needle above the zygoma [13]. These approaches are invasive considering the trauma exerted on the temporal muscle and the scar left in the patient’s scalp.

**Minimally invasive procedures** are advisable when addressing a postoperative complication.

**Endoscopy** has been used through either a trans-sinusal approach or the extraction socket [18]. If such a technique enables appropriate visualization of the tooth, it does not allow tooth stabilization for retrieval.

**Navigation** based on optical tracking has also been described [5]. When available, such an elegant method allows accurate localization but not tooth stabilization. It requires an initial registration step between the CT scan and the patient. As image refreshment is not feasible, the intraoperative activity is based on the preoperatively acquired image data, just like when a standard bite block is used for intraoperative guidance. Any misalignment that might have occurred within the time interval between the CT scan acquisition and the retrieval procedure cannot be addressed. Indeed, mouth opening or instrument position is likely to displace the tooth. Additionally, optical navigation requires that no disruption occurs in the line of sight between patient-borne LED fiducials and the camera. This can be challenging during intraoral procedures with limited operative sight [19].

A PubMed search using the terms “third molar displacement,” “pterygoid fossa,” and “infratemporal fossa” did not retrieve any procedure describing the use of **interventional radiology** for this purpose.

The present report is therefore the first to describe such a **minimally invasive** technique to retrieve a wisdom tooth from ITF. It allowed the swift and safe retrieval because of accurate targeting and steady stabilization.

The rapid and appropriate intraoral placement of the trocar without injuries to the surrounding tissue made surgical retrieval extremely easy and the initially intended **alternative procedures** unnecessary. Indeed, a **snare technique,** commonly used by interventional radiologists to remove vascular foreign bodies, was considered to catch the tooth. It became irrelevant because fibrosis of the surrounding tissue would have probably impeded the deployment of the lasso wire inserted percutaneously through a small needle.

The use of a **cryoablation probe to** generate a small ice ball sticking to the tooth to achieve stabilization and retrieval was also considered [20]. It is currently not recommended and was abandoned, as accidental injury to the cryoprobe (through which high-pressure gases are pushed) is likely to occur in combination with surgery, and this may be lethal for the patient.

The relevance of interventional radiology to retrieve a displaced wisdom tooth can be questioned, especially as the tooth turned out to be retained by fibrosis. In the present report, MSCT used a low-dose protocol and provided better spatial resolution, higher image quality, and fewer artifacts compared to CBCT. We believe that the use of 4D MSCT interventional radiology increases the chance of retrieval success which is of utmost importance when addressing a postoperative complication with potential legal issues.

Should an interventional radiology department not be available in a facility, the patient can potentially be referred to the appropriate unit because tooth retrieval is not an emergency.

Although the cost of the bone trocar is acceptable (110 USD), such a procedure requires two teams of specialists. However, the advent of interventional multimodal hybrid units [21] could allow maxillofacial surgeons to perform such a technique by themselves, making it more cost-effective.

Cost-related concerns are to be balanced with the ease and time efficiency of this nontraumatic procedure resulting in a scarless outcome with an uneventful postoperative course, thereby allowing outpatient care.

In our opinion, such a minimally invasive procedure for wisdom tooth retrieval from the ITF is therefore a treatment option of interest.

## Additional file

Additional file 1: Timeline of the episode of care. (TIFF 1955 kb)

## Data Availability

The datasets generated and/or analysed during the current study are not publicly available due anonymity purposes but are available from the Strasbourg University Hospital on reasonable request.

## Abbreviations

CBCT: Cone beam computed tomography
CT: Computed tomography
ITF: Infratemporal fossa
MSCT: Multi-slice computed tomography
