# Supplementary figures and images for: Retrieving a displaced third molar from the infratemporal fossa: case report of a minimally invasive procedure
Source: BMC Oral Health. 2019 Jul 15;19:149. doi: 10.1186/s12903-019-0852-z (PMC6631546; doi:10.1186/s12903-019-0852-z)

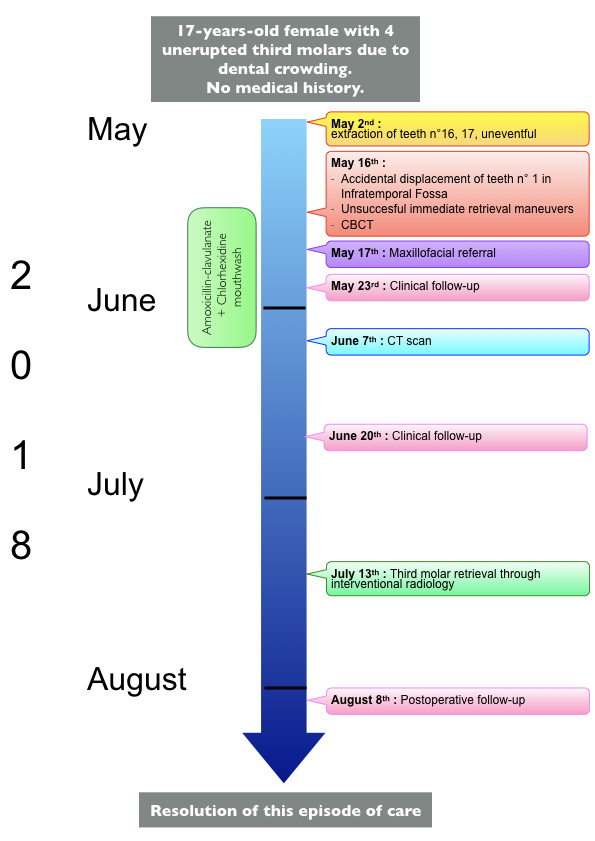

Supplement: Supplementary file 1 — Timeline of the episode of care. (TIFF 1955 kb) [file 12903_2019_852_MOESM1_ESM.tiff]
